# A niche construction-based approach to evolution by natural selection without reproduction—proof of concept and implications

**DOI:** 10.1098/rsos.260737

**Published:** 2026-10-14

**Authors:** François Papale, Yuri Kulish, Joseph Bielawski, Louis-Patrick Haraoui

**Affiliations:** ^1^ Department of Philosophy, McGill University, Montreal, Québec, Canada; ^2^ Department of Biology, Dalhousie University, Halifax, Nova Scotia, Canada; ^3^ Department of Microbiology and Infectious Diseases, Université de Sherbrooke, Sherbrooke, Québec, Canada; ^4^ Centre de recherche Charles-Le Moyne, CISSS Montérégie-Centre, Greenfield Park, Québec, Canada; ^5^ Healthy Microbiomes in a Changing World, Canadian Institute for Advanced Research, Toronto, Ontario, Canada

**Keywords:** evolution by natural selection, reproduction, replication, niche construction, origins of life, autocatalytic sets of chemical reactions, hydrothermal vents

## Abstract

In past decades, conceptual analysis has established that evolution by natural selection (ENS) can occur without reproduction (Bouchard 2008 *Philos. Sci*. **75**, 560–570 (doi:10.1086/594507); Papale, 2021 *Synthese* **198**, 10 429–10 455 (doi:10.1007/s11229-020-02729-6)). The aim of this paper is to provide a computational proof of concept for this theoretical advance and to start assessing some of its conceptual upshots. To do so, we designed an agent-based model that introduces and defends a niche construction-based account of ENS without reproduction (ENSwR, pronounced like ‘answer’). Our results provide a computationally tractable proof of concept for ENSwR and a basis to reflect on some of its conceptual implications for debates regarding the origins of life. First, conceptual analysis suggests that ENSwR provides a novel explanatory framework that supports origins of life theories in which autocatalytic sets (ACSs) of chemical reactions play a central role (Xavier, Kauffman 2022 *Phil. Trans. R. Soc. A* **380**, 20210244 (doi:10.1098/rsta.2021.0244)). Second, through a discussion of the hydrothermal vent theory for the origins of life, we show that ENSwR will contribute to explaining the evolutionary origins of reproduction.

## 1. Introduction

Reproduction is generally assumed to be necessary for evolution by natural selection (ENS) to occur [1–4]. This belief is so well entrenched that the capacity to reproduce is held by many as a defining criterion of life [5]. Recent conceptual analysis, however, has shown that if Darwinian evolution (i.e. evolution that relies on natural selection as a driving factor) is defined as change in the distribution of variation across time that is due to fitness-affecting interactions with the environment (i.e. to natural selection), then populations can undergo Darwinian evolution even if their components, the units of selection, fail to reproduce (or replicate) [6–11]. This insight has the potential to significantly alter the way we think about Darwinian evolution, providing important conceptual advances. In this paper, through a series of simulations, we provide a proof of concept for the theory of ENS *without reproduction* (ENSwR, pronounced like ‘answer’).

Another objective is to start assessing the conceptual upshots of the deep theoretical transformation for evolutionary biology that the move from ENS to ENSwR implies. To do so, we explore how the insights provided by our simulations can inform debates regarding the origins of life, where researchers are faced with situations in which reproduction is notably absent. Our model provides a foundational inquiry into the possibility that microenvironments shaped by niche construction are precursors to entities capable of reproduction. Hence, in addition to providing a proof of concept for ENSwR, we tackle an outstanding question of evolutionary biology: how can reproduction, which is itself an evolved trait, have resulted from Darwinian evolution if Darwinian evolution requires reproduction? Our niche construction-based account of ENSwR provides an answer to this question, integrating both conceptual analysis and modelling.

The paper is structured as follows. In §2, we review the key concepts of ENSwR. This allows us to shed new light on the role of niche construction in Darwinian evolution. In §3, we present an agent-based model that anchors the conceptual framework of ENSwR into computationally tractable simulations. In §4, we show that the theoretical breakthrough allowed by ENSwR strengthens the autocatalytic sets (ACSs) literature [12–17]. Indeed, our niche construction-based approach to ENSwR shows how Darwinian evolution can *operate prior to* and *explain the origin* of ACSs and of reproducing entities.

Throughout this paper, we use the term ‘reproduction’ to refer to processes of multiplication of entities in which parents have a privileged causal input on the traits of their offspring, leading to the presence of vertical lineages linking successive generations [18,19]. We use the term ‘multiplication’ to refer to any process through which entities are being produced; by itself, multiplication entails nothing regarding similarity between producer and products or among products (although some forms of multiplication, such as reproduction, are more restrictive). ‘Replication’ refers to a special case of reproduction, with high-fidelity copying of the entities involved [19–22]. Reproduction is therefore a necessary condition within standard accounts of ENS, whereas replication is facultative [3,18]. ‘Re-production’ refers to cases of multiplication in which entities are produced recurrently without the privileged causal input of similar entities (i.e. without parents) [10].

## 2. Core concepts of evolution by natural selection without lineage formation

Standard approaches define evolution as changes in the distribution of heritable variation within a population across time. While there are debates about how to define Darwinian evolution, it is often considered a more specific process in which changes in the distribution of variation are guided by selective pressures, i.e. the result of interactions between members of a population and their selective environment [2,3,7,18,23,24]. Standard accounts hold that ENS occurs in any population where there is variation related to fitness and *heredity*. Heredity is held to necessitate reproduction, a causal parent–offspring relationship, which ensures that offspring resemble their parent(s) more than they do other units in the population [3,18,25]. Typical examples of reproduction include sexual reproduction, cell division and the templated replication of DNA sequences. More marginal or contested cases include vegetative growth (followed by scission), the recurrent formation of multi-species assemblage (depending on the extent of vertical transmission involved) and clade branching [18].

By contrast (see table 1 for a systematic comparison between standard ENS and ENSwR), the theory of ENSwR suggests that Darwinian evolution occurs in any population where there is variation related to fitness and *memory*. Memory refers to stability in the distribution of variation within a population. The more stable across time is the distribution of variation in a population, the higher is its memory. Hence, just like heredity, it comes in degrees. Too much heredity or memory entails that no new variation is added to the population; too little means that changes in the distribution of variation might end up being random regarding environmental interactions thereby leading to drift. The main difference between the two is that memory is a property of populations, while heredity refers to parent–offspring relationships. Hence, while *heredity* results from mechanisms that ensure the vertical transmission of traits of units of selection, *memory* may result from a more inclusive set of mechanisms that stabilize the distribution of variation *at the level of populations*.

It follows that memory can be sustained by the capacity of units of selection to reproduce (reproduction leads to a certain degree of stability at the population level). Memory and reproduction are nonetheless different concepts. The theory of ENSwR hinges on that distinction: memory can be present even when units of selection fail to reproduce. Our model and simulations illustrate this with the notion of niche construction.

**Table 1.** Differences and similarities between standard ENS and ENSwR.

alternative understandings of Darwinian evolution	standard ENS [3,18,21,26]	ENSwR [7–9,27]
is reproduction necessary?	yes	no, but the population must be regenerated if new traits are to be explained using ENSwR.
Darwinian evolution requires *some* stability in the distribution of variation across time. What sustains this stability?	heredity, itself sustained by mechanisms of vertical transmission of traits of units of selection.	memory, a population trait resulting from mechanisms that ensure some degree of stability in the distribution of variation (whether through vertical transmission of traits or not).
how is fitness defined?	often (but not always) reduced to the reproductive output of a unit of selection; may also include persistence or viability.	a measure of a unit of selection's capacity to increase the ratio of units of selection similar to it in the population (see [28] for details).
in both cases, Darwinian evolution requires …	variation related to differential fitness; fitness entails environmental interactions (natural selection) sufficient population size to avoid drift some degree of stability in the distribution of variation population regeneration to allow small variation to accumulate (required only for origin explanations [18])	

Niche construction is a process of transmission of fitness-enhancing effects across time realized through stable alterations made to the environment by preceding units of selection [29–34]. Beavers, for example, build dams that profoundly alter their ecosystem. Alongside any traits directly transmitted to offspring through genetic inheritance, niche construction by the beaver supports the fitness of beavers in future generations that inherit an environment altered in a beneficial way. Beaver offspring therefore interact with the results of fitness-increasing activities of their ancestors.

Canonical niche construction theory assumes reproduction and builds around it [35]: the beavers reproduce, and their niche constructing behaviour is evolutionarily important *alongside* this reproduction. Through our simulation, we demonstrate that niche construction, i.e. the process of altering the environment in a fitness-increasing way, is relevant even in situations where reproduction is absent. Most importantly, it can accompany other forms of *multiplication* of units of selection. This sheds new light on the importance of niche construction, especially in cases where the environment is the main cause of their multiplication. In the next section, we show that units of selection (e.g. components of ACSs; see §4) can construct their environment in ways that cause the environment to re-produce additional, similar units of selection, satisfying the conditions for Darwinian evolution. In §4, we suggest that this may be tied to the ACS literature and the hydrothermal vents theory for the origins of life. While we are not the first to evoke niche construction at the origins of life [36,37], the originality of our claim lies in the importance of niche construction even in the absence of reproduction and in providing a formally specified agent-based demonstration that this possibility is dynamically consistent.

Before diving into our model, however, some aspects of the concept of niches gain to be reviewed. The concept refers to a subpart of the environment which is analytically partitioned in relation to a given taxon, species or type of interest (see Van Valen's [38] work for a different approach to the concept of niche). In ecology, it is often defined as a multi-dimensional hyperspace where the various dimensions represent environmental conditions that allow for the existence of the population under study. In this paper, we hold a slightly narrower meaning which is typical of how the concept of niche is used in evolutionary biology. We hold a population's (or a species' or a type's) niche to represent the sum of the selective pressures that affect it, either positively or negatively [35,39].

## 3. A model of evolution by natural selection without reproduction driven by niche construction

The theory of ENSwR has sound conceptual bases [6–9,27,40] but lacks a proof of concept. We designed a model to fill this gap. We built an agent-based model to show how niche construction can sustain Darwinian evolution even in the absence of reproduction. We then extended the model in stages to show that strong niche construction by units of selection can overcome differential persistence of competitors. In the final version of the model, we add diffusion of units of selection between microenvironments. This provides a foundational inquiry into the possibility that microenvironments shaped by niche construction are precursors to entities capable of reproduction.

Agent-based modelling is a well-established practice in evolutionary biology and across the sciences, enabling researchers to study how complex system-levels emerge from interactions among simple, discrete units. These countable units in the model are *causal* units in the sense that things happen to them, and they cause things to happen in the system as well. These causal relationships are entirely encoded in the program that drives the simulations. These specifications are important because the word ‘agent’ is used, in this paper, differently from how it is used in disciplines such as philosophy of biology. Neither goal-directedness nor purposefulness is implied.

The model is a general one, abstracting away idiosyncrasies of specific evolutionary scenarios to focus on core dynamics. As such, it is *not* meant to capture with precision the chemical phenomena we discuss in §4. Our model aims to establish a proof of concept for ENSwR, and it does so by drawing on modelling practices well entrenched in evolutionary biology. For instance, our model is concerned with change in the distribution of variation within a population, but no mutations are added to the population across time. This is in line with the tradition of modelling the effect of natural selection on *existing* variation. As per Godfrey-Smith [18], this is a focus on distribution explanations, which are the most fundamental Darwinian explanations (because they are the basis for what he calls origin explanations, that is, explanations of adaptations). All of this means, however, that our units of selection feature no macro-evolvability. ENSwR would benefit from further modelling that allows units of selection to accumulate variation across time. Our model offers the groundwork for that and more broadly to develop a research programme based on ENSwR.

### 3.1. The basic model

We provide the most important information about the model here, but more details can be found in the supplementary material. A repository containing the code and results discussed in this paper is publicly available [41].

Our model features a spatial grid of 10 by 10 microenvironments (although simulations presented in §§3.2 and 3.3 focus on dynamics occurring within a single microenvironment). The units of selection, in this model, have different properties related to (i) the rate of production by the local microenvironment, (ii) the decay rate, and (iii) the capacity to modify the environment. In our simulations, we track two types of units of selection, denoted by A and B. Units of both types in a microenvironment form a single (physically bounded) population.

At regular time intervals, all A's and B's are terminated. These ‘extinction’ events, which take up a time step in our simulations, represent occasions when regional environmental conditions exceed the physical tolerance of A and B. An example of empirical situations that echo this feature of the model are palaeosterilization events [42], which are especially relevant in the contexts discussed in §4. In hydrothermal vents, for instance, periodic flows of hydrothermal fluids that exceed the temperature that can be tolerated by local entities are an example of palaeosterilization [43]. For the purpose of our model, the term *palaeoextinction* is more appropriate.

Extinction of all A and B units serves two purposes in our model: (i) it blocks the material distribution of A and B from directly affecting the future distribution of variation within microenvironments, leaving only their imprint on the environment to affect future distribution of variation, and (ii) it mimics the profound instability expected of origin-of-life settings (e.g. palaeoextinction), setting the stage for the discussion in §4. The first point strengthens the case for ENSwR, making it impossible to reduce the dynamics at work to reproduction with vertical transmission.

Indeed, the central idea that drives our model is that A and B have the potential to interact with their environment to bias future production of A and B units locally, even if they do not reproduce. Each type of unit carries a baseline production rate (the realization of units in the environment through a random process) and is also attributed a value that translates its niche construction capacity. When an individual unit is introduced in a microenvironment, its niche construction value influences that microenvironment. Future production of units in that environment is a function of niche construction effects and production rate. In other words, when units with niche constructing potential are added to a microenvironment, they increase the chances that more units of the same type are produced in the future. The additive effect of niche construction lasts beyond the periodical extinction of A and B. Note also that the influence of A's and the influence of B's on their environment are modelled independently of one another, but that it influences production of both A's and B's in cases with limited resources (see below). This operationalizes the notion of niche construction in a context without reproduction, as conceptualized in the previous section. It also means that the environment, in our model, is an active component of the causal processes at play, being both affected causally by the units and a driver of the selection process (see especially §3.3 on persistence).

This, however, contrasts with paradigmatic cases of niche construction, e.g. a beaver building a dam. In such cases, an individual unit of selection can niche construct its environment in a way that leads to direct influence on fitness. Sterelny [44] uses the expression ‘ecological engineer’ to describe units of selection that realize paradigmatic niche construction. He contrasts these with collective niche constructors that fail to reap direct harvests, giving the example of litter-eaters. These, he argues, cannot be selected for their niche construction because the effect on their fitness is too remote and based on collective efforts. Our units of selection combine elements of paradigmatic and collective niche construction: the fitness of a type is directly increased through niche construction, but this *requires* the collective input of many units. Yet as our simulations show, this allows for the better niche constructors to be selected despite the absence of reproduction.

It is important to note that some aspects of the concept of niche are somewhat obscured in our model. More precisely, (i) the multi-dimensionality of niches is partly blackboxed and (ii) the dependence of niches on specific populations is absent because we have a single population of interest. Regarding (i), our model presents the units-environment relationship with only three main dimensions: production rates of units, persistence and niche construction effects. However, this is the result of a simplification process that is necessary for the tractability of the model. We should assume, for instance, that persistence rates are best understood as resulting from highly multi-dimensional relationships between units and their environment, but in our model, the complexity of ecological interactions that affect persistence is collapsed into a single variable.

Regarding (ii), it must be noted that both types of units (A's and B's) present in the model form a single population and that we focus on the parts of their respective niches that overlap (assuming that the same resources are necessary for their production). This may give the impression that their effect on the environment cannot be described as niche construction. We believe it is best to describe it as such for at least two reasons. First, the process here described could lead to cumulative evolution, and thereby to the divergence of the two types. As a result, their niche would become increasingly distinct. As with (i), it is only the simplifications required for our proof of concept that give the impression that the population-specificity of the niche concept is absent. Second, in a population with competing variants that hold very similar niches, it is usually expected that one will exclude the other. By contrast, our model suggests that such a population may, under certain conditions, reach equilibrium in a state where both types are present (fig. A1 supplementary material). This equilibrium is therefore best represented as a form of polymorphism, which is compatible with the fact that the two variants have overlapping niches.

Taken together, these clarifications warrant seeing the effect of units on their environment as a type of niche construction. The niche construction here entails that the units actively alter the set of selective pressures that influence their evolution.

We developed two versions of the model: one with unlimited resources available for production of units and the other with a fixed limit on those resources. The main difference between limited and unlimited resources versions of the model is that in the latter, the production rates of the types are independent of one another. In the version where resources are limited, increased production of A's means a decreased production of B's (and vice versa) because their production depends on the shared pool of resources. In cases with a limited number of resources, the production of units of selection is modelled as a Bernoulli experiment occurring each time step (eqn 2 in the supplementary material). In these experiments, consumption of each resource is a trial where the probability of the outcome is determined using both the starting production rate and the accumulated effect of past niche construction. In this sense, niche construction of A's affects production of B's also and vice versa. In §3.2, we compare cases with both limited and unlimited resources. In §§3.3 and 3.4, we focus on scenarios with limited resources to explore the interplay between niche construction, differential persistence and diffusion across a metapopulation.

Finally, we note that, in this model, time is broken down into discrete time steps. These time steps are based on the environmental production rate of units of selection, whereas time in many evolutionary models reflects biological reproduction rates instead. For simplicity, we assume that A's and B's require the same amount of time between production events, such that we have unified time steps. We also simplified the model by assuming that production events are discrete rather than continuous.

### 3.2. Niche construction versus differential production rates

Our first simulations are meant to establish that niche construction increases the ratio of the niche constructing units despite stochastic events such as extinctions (note that we modelled regular extinction events to aid visualization). The objective is to compare the long-term outcome of niche construction to either a high starting production rate or a mix of high starting production rate and moderate niche construction (figure 1). In this design, type A is always better at niche construction than B, and type B starts with a higher production rate (twice that of A in each simulation). We also compared outcomes under unlimited (figure 1a,b) and limited resources (figure 1c,d). Figure 1a,c provides results of simulations where A's have low production rates but modify the local environment in a way that is favourable to their re-production in the long run (A's niche construction value is 2). Figure 1b,d provides results of simulations where B's also have the capacity to alter the local environment over the long run, but less effectively than A's (B's niche construction value is 1). In all cases, A's come to dominate the population distribution through niche construction.

**Figure 1.**
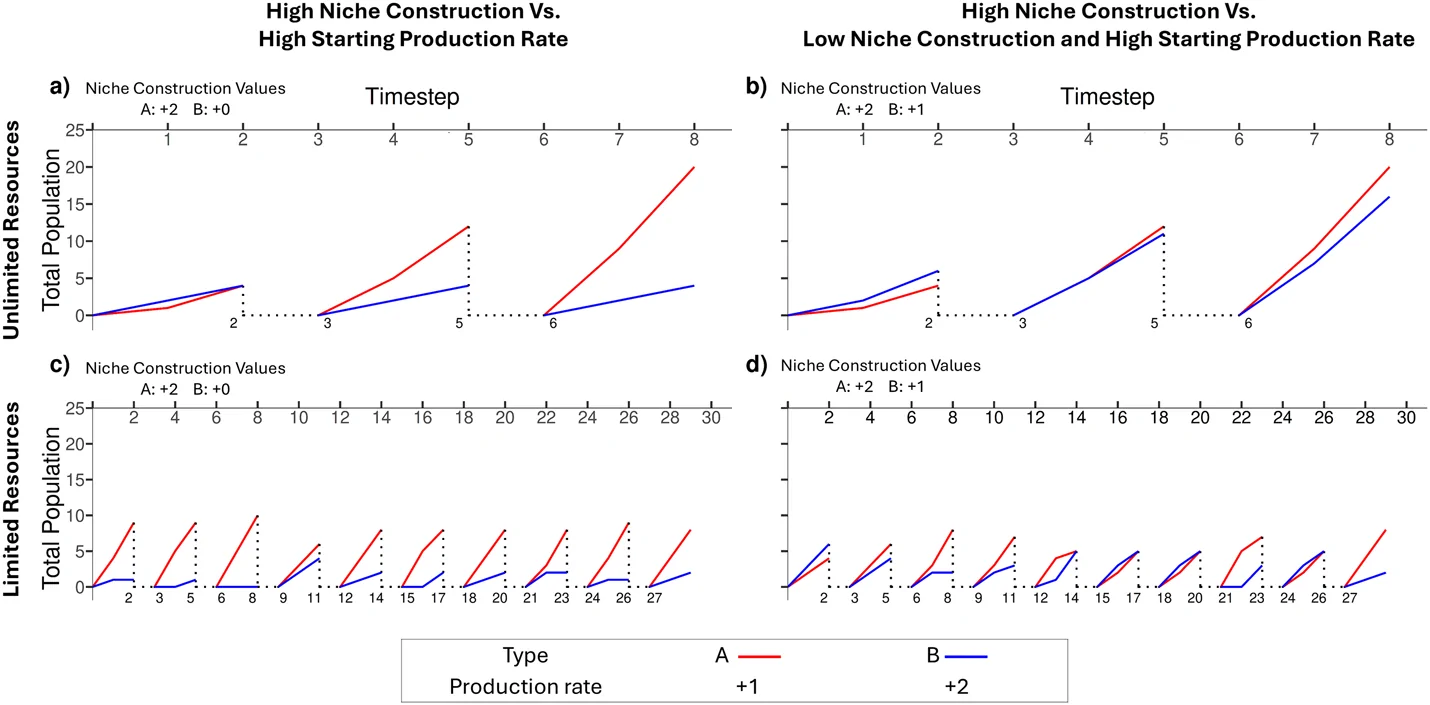
Population dynamics with differential niche construction and differential production rates. This figure illustrates simulations done with two time steps separating each extinction event (extinction events also count as time steps) and with the production rates favouring B's. Extinction events bring the populations down to zero (the dashed lines represent continuity only in the sense that the dynamics happens in the same microenvironment across extinction events). Figure 1a,b presents scenarios with unlimited resources, while figure 1c,d presents scenarios with limited resources. In figure 1a,c, only A's niche construct. In figure 1b,d, B's can also niche construct, but A's do so twice as well as B's.

Some differences must nonetheless be noted. When only A's niche construct, the relative distribution of A's in the population reaches 83% when resources are unlimited after only two extinction events (the situation is exacerbated when resources are limited). The dynamics is more complex when B's also niche construct. In both scenarios (limited and unlimited resources), species B initially dominates the population, and under limited resources, it takes more than a dozen extinction events for A's to reach a frequency comparable to the one they achieve quickly in figure 1a,c (figure 1d: A's reach 80% of the distribution before the tenth extinction event).

These very basic simulations serve as proof of concept for ENSwR: it shows that change in the relative frequency of A and B results from environmental interactions that feedback as differential multiplication of A's and B's within the environment. Recall that A and B do not form lineages (individuals have no privileged causal effect on the production of specific units of A and B in the future) and undergo periodic ‘mass extinctions’ removing all existing units, entailing complete regeneration of populations. Nevertheless, the dynamics of this simple case, which embodies ENSwR, corresponds to Darwinian evolution. Indeed, both ENS, which entails reproduction and heritability, and ENSwR can be concisely defined as changes in the relative frequency of traits in a population owing to natural selection (i.e. selective pressures resulting in differential fitness) [9,18,24]. Here, the relative fitness of A and B is expressed as the expected change in the ratio of each in the population, as a result of interactions with the environment (in the form of both niche construction and resource consumption). This corresponds to the use of fitness in population genetics (§4.1). In the next round of simulations, we add differential persistence to the model, situating our approach more firmly into standard evolutionary modelling practices.

It could be argued that in situations with limited resources, the effect size of competition for resources between individuals might render the effect of niche construction irrelevant. In our model, this is not the case. First, recall that while production rates are encoded as traits of types, the production is nonetheless postulated to be realized by the environment. Furthermore, this production is mediated by the aggregate effect of niche construction. This aggregate value is a trait of microenvironments, not of units of selection. Second, in figure 1c,d, there is indeed competition between A and B, at every time step, to use the limited resources available for unit production. The effect of resource consumption is strongest at the start because niche construction has not yet affected the baseline production rate of the microenvironment. Had the results been entirely driven by competition for finite resources, the type with the highest production rate would always dominate the population distribution. Our results show that an initial advantage in production rates can be overcome by niche construction. Note that results derived from simulations with unlimited resources can only be attributed to the beneficial effect of niche construction on the relative frequency of A (figure 1a,b). Those same processes also determine the outcome when resources are limited, although a greater number of time steps is needed owing to competition for resources modulating the effect-size of niche construction (figure 1c,d). Note also that we use a relatively low quantity of resources in limited-resources scenarios to limit the required compute time. Increased resources would probably favour type A. Having established this proof of concept, the rest of the results are generated under limited resources (where there is an interplay between competition and niche construction), as this better reflects potential *in situ* conditions where competition for resources is relevant.

### 3.3. Complexifying the model: differential persistence

In the simplest versions of the model (§3.2), there is no differential persistence (i.e. A's and B's have the same decay rate, lasting until the next mass extinction event), such that niche construction and production rates are the only potential features that can lead to change in the distribution of variation. Here, we introduce a decay rate for each species, which has no effect on available resources. At each time step between extinction events, individual A's and B's are removed from the population according to their decay rate. To allow decay rates to influence the dynamics of the population, we extended the extinction intervals from two time steps (in the previous section) to 50 time steps.

First, we provide a null situation where population dynamics is driven only by differential persistence. B's are the better persistors; A's decay after only 10 time steps, while B's decay after 20. Figure 2a shows that in this situation, B's dominate the population following each extinction event. Figure 2a illustrates that it takes approximately 20 time steps to reach predictable equilibrium dynamics after each extinction event. The equilibrium where B's dominate over A's is simply a consequence of individual B molecules having a longer residence time owing to their lower decay rate as compared to A's. Note that, in contrast to figure 1, the baseline production rates for A and B are the same throughout figure 2. Thus, comparisons with the null case (figure 2a) throughout figure 2 reveal the interaction between decay rate and niche construction.

**Figure 2.**
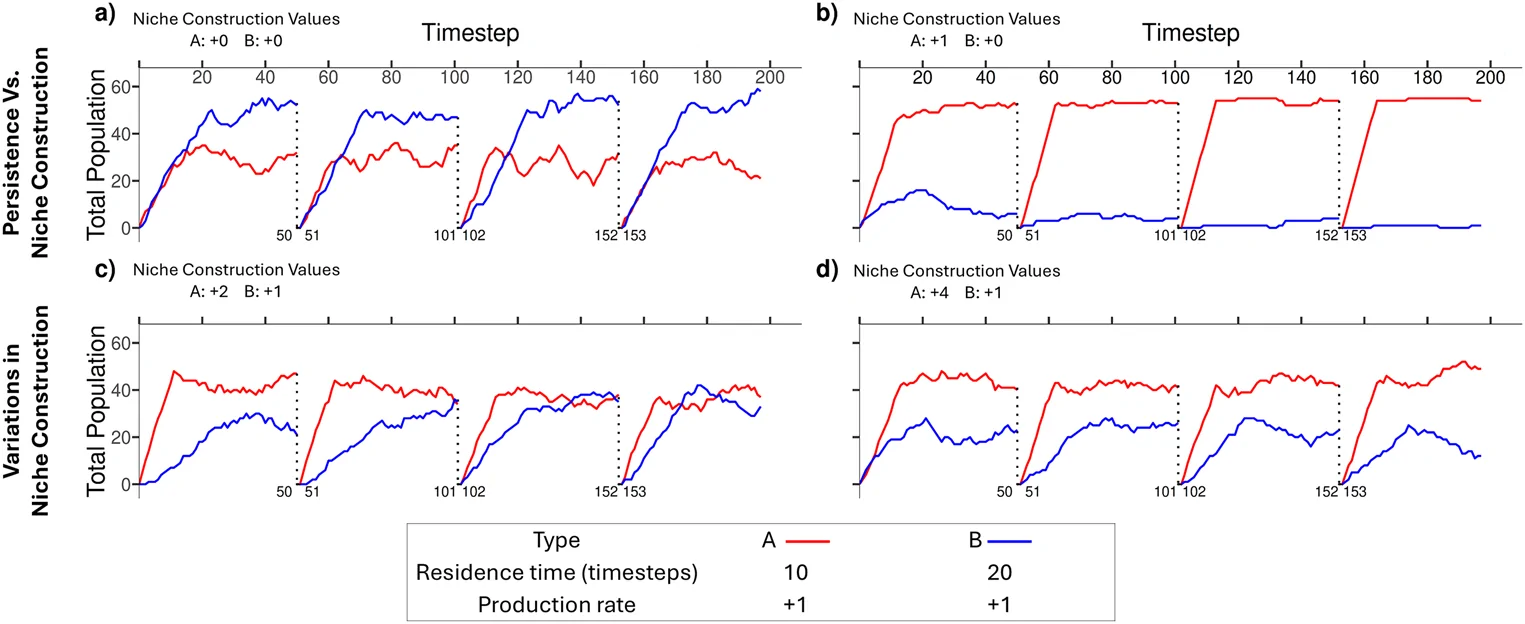
Differential niche construction, differential persistence and extinction-event frequencies. This figure illustrates the dynamics within a single microenvironment in cases where there is differential persistence as well as differential niche construction. Specifically, B's persist twice as long as A's throughout the figure. In figure 2b, A's niche construction is introduced. In figure 2c,d, A's and B's are niche constructors: there is differential persistence and differential niche construction.

In figure 2b, we re-introduce niche construction. Here, B's still have better persistence than A's (20 versus 10 time steps), but A's are now capable of niche construction. As expected, A's very quickly come to dominate the population (as in figure 1a,c). This implies that a niche constructing unit of selection with the capacity to alter its environment in a way that favours its future production can dominate in a population where competitors are more stable (that is, they decay less quickly).

Figure 2c,d explores the relationship between persistence and niche construction in cases where both competitors (A and B) are niche constructors. In figure 2c, B's survive again twice as long as A's, but A's niche construction is twice as efficient. In this situation, the population reaches equilibrium with similar quantities of A's and B's present. In this scenario, neither A's niche constructing advantage nor B's better persistence is enough to make one of the two types take over the population. By contrast, figure 2d illustrates that doubling the niche constructing capacity of A's causes them to dominate the population even before the first extinction event, with subsequent extinction events increasing the difference. Figure 2c,d demonstrates that the degree to which better niche constructors dominate a population depends on the interaction with decay rate. A stronger niche construction capacity is required when a competitor has a longer residence time owing to a low decay rate. In other words, even though niche construction is an inherent advantage, its effect size can be matched by other contextual variables, such as the decay rate of units of selection, the decay rate of the niche constructed environment or time intervals between extinction events (the latter two would gain to be studied in future work).

### 3.4. Niche construction and diffusion

For this last series of simulations, we added diffusion across a metapopulation (something akin to migration [45]) in order to evaluate the potential of niche constructors to spread beyond their local microenvironment (imagine a network of rocky pores, or compartments in a hydrothermal vent, between which local diffusion of chemical species (potential units of selection) is possible at a low rate). While chemical diffusion has been used as a basis to understand movement of organisms within a population before [46], here we use the word ‘diffusion’ simply to account for the partially randomized movement of units of selection from a microenvironment to neighbouring ones.

The simulations were conducted using a 10 by 10 grid of microenvironments, with border cells linked (so that there is no inherent disadvantage to being produced in a ‘corner’ microenvironment). At the start of simulations, only one microenvironment produces A's (this is meant to be a worst-case scenario for A's and their capacity to spread across the grid). As before, both types must compete for limited resources for production. During each extinction event, occurring every 20 time steps, individual units of selection of both types have 1% chance to diffuse from a microenvironment to a *neighbouring* one instead of being completely removed from the metapopulation system. If a niche constructor diffuses to an adjacent microenvironment, its additive effect on production rates (i.e. its niche construction value) is applied to the new environment (without removing its effect on its original microenvironment).

In the scenario depicted in figure 3, A's are niche constructors whereas B's lack the capacity to niche construct. However, B's have better persistence (20 time steps) than A's (10 time steps). As a result, despite A's only being produced in a single microenvironment at the start of the simulation, they come to dominate the distribution of variation across the metapopulation after 20 extinction events, and they reach near fixation after 35 (note that we describe the dynamics using extinction events, as the time scales involved are much longer than those relevant in the scenarios depicted by figures 1 and 2). This result is consistent with our previous finding that niche constructors can dominate competitors that persist longer, while extending the result to include the capacity to dominate across a metapopulation when diffusion is possible. Once again, the simplicity of the dynamics involved contributes to the clarity of the proof of concept that this model is meant to provide for ENSwR: evolutionary dynamics occurs in a Darwinian fashion despite the absence of reproduction.

**Figure 3.**
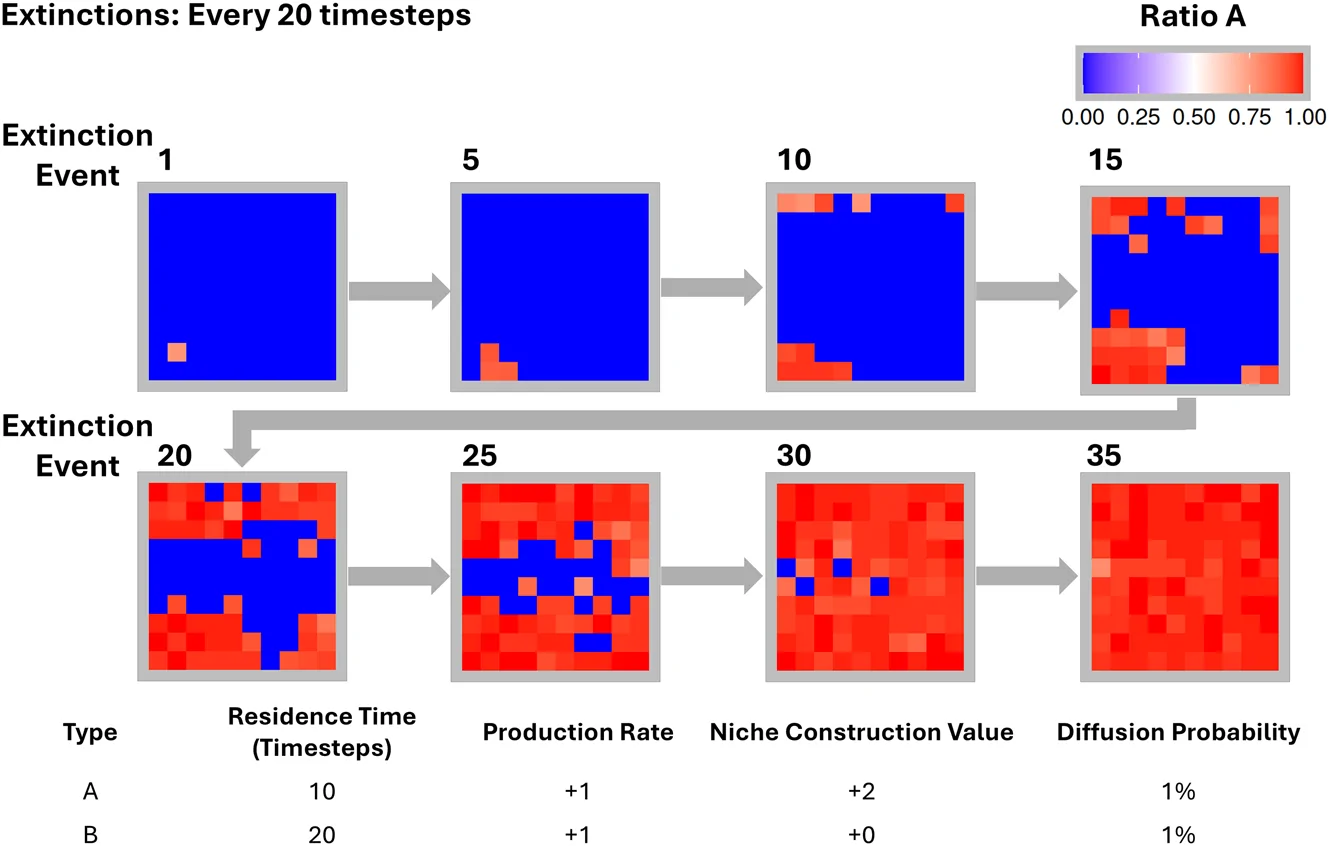
Spread of niche constructors across a metapopulation through diffusion. This simulation introduces the diffusion of units across microenvironments. During each extinction event, individual units of selection have a 1% chance of being sent from one microenvironment to a neighbouring one instead of being removed from the metapopulation. When this happens, the niche construction effect of the molecule affects its new microenvironment. The ratio of A's and B's represented by the heat maps is that observed during the last time step before the indicated extinction event.

Finally, metapopulation dynamics depends on the strength of the interaction between niche construction and persistence (see fig. A1 in the supplementary material). Fixation (of A's) only happens when the effect size of niche construction is sufficient to overwhelm that of other traits such as persistence. Niche construction is advantageous, but not overwhelmingly so, such that an equilibrium between A's and B's can be reached across the metapopulation.

## 4. Discussion: some conceptual upshots of evolution by natural selection without reproduction

While §2 reviews the basic concepts of ENSwR and sheds new light on the importance of niche construction for evolutionary dynamics, §3 provides the first simulation-based validation of this groundbreaking approach to Darwinian evolution. Uptake of ENSwR by the community will have many effects on evolutionary biology, beyond that which can be discussed in a single paper. For example, holobionts, multi-species biofilms, ecosystems and cultural phenomena share a common feature: their fitness is sustained in part by traits that are *not* heritable. For this reason, we anticipate that ENSwR will have broad applicability. Here, we wish to explore the conceptual consequences of ENSwR for a specific field, namely, origins of life research. To do so, we first discuss an ACS-based interpretation of our model, which leads us to discuss the notion of fitness. Second, we emphasize the potential contribution of ENSwR to origins of life debates through coordination with hydrothermal vents-based theories. This work paves the way for empirical inquiries guided by ENSwR.

### 4.1. Evolution by natural selection without reproduction, fitness and autocatalytic sets

ACSs are sets of reactions in which each reaction is catalysed by some of the products generated in the set. While ACSs mostly refer to sets of *chemical* reactions, the framework is now used to tackle a broader range of networks [17,47,48]. We believe that the theory of ENSwR strengthens the evolutionary framework surrounding ACSs, especially at the origins of life. Specifically, we suggest that in certain cases, the focus should be on *components* of ACSs as units of selection rather than on ACSs themselves. However, because these components do *not* reproduce, this raises an important set of questions: how can these components be said to be fit, how can their fitness be measured? The concept of fitness, like many other concepts in evolutionary theory, has been designed to account for cases where reproduction is present. A simple reasoning by abstraction allows us to apply this concept to ENSwR and to ACS components while conserving its essence, namely, the idea that a unit's evolutionary ‘success’ depends on interactions with the environment and its capacity to influence the ratio of similar units in the population.

The notion of fitness is often defined in terms of reproduction [18,28,49–51]. But reproductive output represents only one proxy for fitness and obscures the multiple mechanisms through which units of selection contribute to future populations, including survival, differential life-history performance and, we argue, niche construction [27]. In population genetics, fitness is treated as a standardized statistical quantity, defined so as to predict changes in population composition under natural selection [52–55]. Standardization starts with absolute fitness. Absolute fitness of a ‘type’ combines *viability* (obtained by dividing the number of individuals of a type *after* selection by the number of individuals *before* selection) with the fertility of that type (obtained by dividing the number of descendants by the number of parents *after viability selection*). Relative fitness converts absolute fitness into a measure of ‘performance’ relative to the other types in the population, typically through normalization. This means that the choice of fitness measure does not merely summarize evolutionary outcomes; it also determines which kinds of causal contributions are recognized and which are systematically standardized away.

Abstracting away the specific means through which units can be said to be *fit*, fitness is simply the capacity to influence the ratio of entities with the same or similar trait in the population, which must result from their interaction with the environment [56]. Other ways to define fitness are special cases of this more general formulation. Indeed, reproduction is a specific way by which a unit can increase the ratio of entities with similar traits in the population, but other alternatives also match the notion's conceptual importance in a Darwinian context. For instance, the effect of niche construction on the relative frequency of a type could be weighed in the above measurement procedure just like fertility is; it could even replace fertility altogether and it certainly upholds the importance of unit-environment interactions. This would still allow predicting population dynamics based on fitness values. In other words, removing reproduction from the *definition* of fitness provides a more general concept that preserves its original conceptual role within the Darwinian framework, but also expands it to cases where reproduction is absent.

This generalization of fitness aligns with ENSwR and opens important conceptual doors. Regarding ACSs, downplaying the importance of reproduction allows for *components* of ACSs to be conceived of as units of selection, i.e. fitness bearers [27], even though they are incapable of reproduction (they rely on the network for their catalysis). By generating a network that increases their production rate, they causally contribute to improving the frequency of similar entities in the population. This is one way of interpreting our simulations that is compatible with the ACS literature [13,14,17].

Our model is also very similar to that of Hordijk *et al.* [13] for chemical reactions in a compartmentalized setting. The appearance of the first components of ACSs, in their model, is a stochastic process, but once the units spark an autocatalytic network (i.e. when the units niche construct), the rate at which they are being re-produced is *not* left to chance anymore. In this light, the niche constructing molecules are fit; they are units of selection. This is a reinterpretation of Hordijk *et al.*’s [13] work based on ENSwR: as is the case with most of the literature on ACS evolution, their paper focuses on ACSs as units of selection; the potential of their components as units of selection remains unexplored. ENSwR provides the framework for exploiting this underappreciated evolutionary phenomenon, something that is especially important at the origins of life.

### 4.2. Evolution by natural selection without reproduction, hydrothermal vents and the origins of reproduction

Prebiotic evolution is often assumed to require a new theory. For instance, Williamson recently suggested that Darwinian evolution is incompatible with prebiotic chemistry, and that a new evolutionary mechanism is therefore needed [57]. The feeling that prebiotic evolution requires an alternative to Darwinian evolution is widespread [58–62] but leans heavily on the belief that the latter requires reproduction [63–65] and genetic material [66]. Our simulations show that Williamson's insights regarding chemical entities indirectly increasing their presence in a population actually sit well with Darwinian evolution as conceptualized by ENSwR. Indeed, in our words, what Williamson emphasizes is the phenomenon of niche construction as operationalized above. Yet he presents it as incompatible with Darwinian evolution. By contrast, our model demonstrates that niche construction can drive Darwinian evolution even in the absence of reproduction and genetic material, thereby extending to forms of chemical evolution such as those described by Williamson. This leads us to argue, in what follows, that ENSwR strengthens a specific approach to prebiotic evolution, i.e. the theories centred on hydrothermal vents.

Hydrothermal vents are seafloor structures, such as the Lost City hydrothermal vents [67], whose compositions and topologies are of special geochemical interest for origins of life research. Namely, the rocky formations present far from equilibrium conditions with various gradients enabling and maintaining organic chemistry, such as pH and redox potential gradients [68–71]. These gradients result from the sustained contact of slightly acidic (Hadean) ocean water and alkaline H_2_ rich hydrothermal fluids [68,72]. The thermodynamic and chemical conditions in such settings can lead to the precipitation of (mainly) FeS and Fe(OH)_2_ [73] that then forms a ferrous layer between the two fluids (an inorganic membrane). The formation of this layer is a crucial step in the development of positive feedback loops that maintain and even strengthen the gradients that gave rise to it [73–76]. This leads to the formation of rocky pores (i.e. compartments) that have been hypothesized to provide conditions in which ACSs involving prebiotic molecules could arise. That is the heart of the hydrothermal vents theory for the origins of life.

This theory fits in the broad framework of ENSwR. From the perspective of individual molecules (e.g. acetate or pyruvate [77]) involved in prebiological ACSs, this is a phenomenon of niche construction as described in the previous section: by transforming their environment (i.e. by contributing to an ACS), the molecules ensure the proliferation of their type (i.e. increase their relative frequency [57]) despite being incapable of reproduction (i.e. these chemical species cannot form lineages). Hence, our model could serve as a basis for studying evolutionary phenomena taking place in hydrothermal vents before reproduction and genetic material while still embracing Darwinian evolution.

Because of this, ENSwR could fill an important gap in origins of life theorizing. The hydrothermal vent theory, as well as ACS literature more broadly, takes for granted the notion of ACS reproduction. Reproduction being a process of *multiplication*, ACS growth and maintenance *are insufficient* to account for ACS reproduction (because they do not, by themselves, lead to multiplication). Our model provides a specific mechanism for explaining how the transition from ACSs persistence and growth to ACS multiplication may occur. In figure 3, the spread of A's can also be interpreted as the spread of A-dominated microenvironments. This means that A-dominated microenvironments are being *multiplied*. While we are far from a case of bona fide reproduction like cell division, this scenario nonetheless brings to mind multi-level selection [21,78], involving both the level of ACS components and the level of the ACSs they give rise to. This could lead to proper evolutionary explanations of the origins of ACS multiplication.

This rationale could strengthen the hydrothermal vents theory. Harrison *et al.*’s [77] recent review of hydrothermal vents theories, for instance, offers no discussion of how protocells (complex ACSs) could, in order to undergo division, free themselves of the inorganic membrane that they relied upon to grow. Their rationale suggests that the result of an evolutionary process, i.e. the emergence of protocell-level reproduction, is taken for granted. Microenvironments with characteristics that result from niche construction in hydrothermal pores could very well be precursors to reproducing entities, but this requires explaining. ENSwR can address this blind spot by providing the basis for an evolutionary explanation of the origins of ACS multiplication through component migration.

This leads to a different outlook on protocells qua reproducers but also, by extension, on the biological entities that followed. While evolutionary dynamics was clearly altered once the first cells started reproducing with heredity [79], the early-life equivalent of what are now cells were microenvironments being shaped by the niche construction effects of chemical species. We believe that before being units of selection in their own right, precursors to cells were something like ecosystems. This claim strengthens the expanding literature surrounding ACSs within and beyond the origins of life research [17,47,48]. Moreover, this may warrant conceiving of cells and organisms, to this day, as being niches (or evosystems [80]) as much as they are ‘individuals’ or units of selection.

## 5. Concluding remarks: generalizing our results and evolution by natural selection without reproduction

The Darwinian theory of evolution has outstanding explanatory power and provides grounds for exploring the history and dynamics of biological evolution, but it has historically been focused on lineage-forming entities. This paper expands the reach of Darwinian evolution. It offers a conceptual apparatus (§§2 and 4) and a proof of concept (§3) that support ENSwR, a version of Darwin's theory that is free of the unwarranted yet pervasive metaphysical assumption according to which units of selection should be reproducers or replicators.

By modelling ENSwR with niche construction, this paper provides bases for exploring previously underappreciated evolutionary phenomena in a way that contributes to the perennial challenge of integrating evolution and ecological theories more thoroughly [81,82]. A variety of biological entities (holobionts, ecosystems, genomes, etc.) as well as unsuspected protobiological (catalyst molecules, microenvironments, etc.) and epibiological (social institutions, corporations, etc.) entities may form populations that evolve by natural selection. Furthermore, our approach, by linking niche construction and ACSs explicitly, strengthens the intuition that environmental scaffolds guiding evolution might be more important to the origins of life than the production of specific molecules (e.g. DNA) [80,83–85].

Among the many fields in biology that will benefit from the maturation of ENSwR, we explored the theory's effect on origins of life research. Specifically, our model solves some of the theoretical problems faced by origins of life literature. Indeed, we have discussed how, in theory, prebiotic chemistry could sustain Darwinian evolution even in the absence of reproduction. We also highlighted that ENSwR offers a preliminary explanation of the evolutionary origins of reproduction. Our work thereby offers conceptual tools to bridge the gap between chemical and biological evolution in a way that warrants pushing the Darwinian threshold [79] much further back in time than where it has historically been set: while the Darwinian threshold was initially tied to the emergence of reproduction with vertical inheritance, we believe prebiotic chemistry, in certain contexts, may have had all the necessary ingredients for Darwinian evolution to occur. Importantly, all these conceptual advances were achieved without developing an alternative to the Darwinian framework, as ENSwR is embedded within evolutionary biology's overarching theory.

## Supplementary Material



## Data Availability

The code used for our simulations is publicly available [41]. Supplementary material is available online [86].
