# Supplementary material for: A niche construction-based approach to evolution by natural selection without reproduction—proof of concept and implications
Source: R Soc Open Sci. 2026 Oct 14;13(10):260737. doi: 10.1098/rsos.260737 (PMC13682428; doi:10.1098/rsos.260737)
Supplement: Supplementary file 1 [file rsos.260737.zip › rsos.260737/rsos260737_si_001.docx]

**APPENDIX**

Simulations were performed using an agent-based model with timestep progression programmed in Python 3. A repository containing the code and results discussed in the paper is publicly available (reference 39 in main manuscript). This model simulates interactions between two key entities: units of selection belonging to one of two types and a grid divided into local environments (grid cells). Different types represent units with different baseline production rates. The continuous process of production is discretized into timesteps within the model. Furthermore, units of each type can modify their local environment to promote the production of more units of the same type. As such, local environments possess properties conducive to each type, and local environments can be modified when a unit is introduced in that environment. These modifications are implemented in the model through the impact of a unit on the value of “niche property” in its local environment. Periodically, the environment suffers a ‘mass-extinction’ during a timestep. Such an event causes all units to be removed from the whole environmental space (i.e., flushed or destroyed). However, the local environments are not destroyed in this process, allowing prior modifications of the local environments to persist as production is re-established.

This model has independent settings to enable or disable resource limitation, particle decay, and migration conditions. Using combinations of these conditions, we can create eight ($2^{3}$) unique modes of simulation.

**Unit production under unlimited resources**

When resources are unlimited, creation of units of a given type is determined by the sum of its type-specific production rate and the niche property of the local environment (Table A1*,* Equation 1). In this scenario, production of a unit type does not affect the production of the other type, and there is no imposed maximum limit on production for any type.

$\# of X Units produced= X_{production\_rate}+{Local Environment}_{X\_niche\_property}$	(1)

**Unit production under limited resources**

In this scenario, the production of a unit is limited by the availability of resources. These are handled within the model by a generalized “resources” parameter. Resource utilization is modeled independently within each local environment. In our simulations, each local environment replenishes to a fixed number of resource “doses” (i.e. 5) every timestep, which reflects the maximum production limit within the local environment. Each dose of resource is used to produce a unit of a specified type during each non-extinction timestep. This process is modeled as a Bernoulli experiment occurring each timestep. In these experiments, consumption of each resource “dose” is a trial where the probability of outcomes can be determined by the accumulated effects of a type’s baseline production rate and the niche-constructed properties of the local environment (Equation 2). This imposes resource competition for production of units of different types.

For each Bernoulli trial at grid cell *n, m*	(2)

${P\left( A \right)}_{n,m}=\frac{A_{production\_rate}+{A\_niche\_property}_{n,m}}{\left( A_{production\_rate}+{A\_niche\_property}_{n,m} \right)+\left( B_{production\_rate}+{B\_niche\_property}_{n,m} \right)}$

${P\left( B \right)}_{n,m}=\frac{B_{production\_rate}+{B\_niche\_property}_{n,m}}{\left( A_{production\_rate}+{A\_niche\_property}_{n,m} \right)+\left( B_{production\_rate}+{B\_niche\_property}_{n,m} \right)}$

${P\left( A \right)}_{n,m}+{P\left( B \right)}_{n,m}=1$

**Niche Construction**

Niches in this simulation are assumed to be unique to their supported type without overlaps or exclusions. While implementation of niche construction is agnostic to resources and potential competition, the niche impact of an individual niche constructor diminishes proportionally to the environment’s existing niche-property value (Equation 3). This prevents compounding exponential growth and asserts the assumption that “well-developed” niches become harder to improve.

(3)

$${Individual impact}_{t} =\left\{ \begin{aligned} X_{niche\_construction}, &{X\_niche\_property}_{n,m,t}=0 \\ \frac{\begin{aligned} \\ X_{niche\_construction} \end{aligned}}{{X\_niche\_property}_{n,m,t}}, &{X\_niche\_property}_{n,m,t}>0 \end{aligned} \right.$$

**Particle Decay**

Decay reflects the fact that some units are more stable than others and that they therefore persist longer. In these models, types have a residence time parameter which specifies how many timesteps a unit of that type persists. When a unit has exceeded its residence time, it is considered to have decayed, and it is removed from the environment. Without decay, units persist until they are destroyed in a mass-extinction timestep.

**Diffusion (an analogue to “migration”)**

Chemical diffusion is well understood, and because it is mathematically convenient, it is also often used as a model to approximate large scale patterns of animal movement. Here we are looking at something else, i.e., movement of units caused by something like a mechanical force in the environment. We call this “particle diffusion” simply to refer to the partially random movement of our units across the environment. We implement diffusion via a random process of unit movement between neighbouring local environments (grid cells). In this model, unit movement is coordinated by the SimEngine at discrete time points (*i.e*., we discretized and marginalized the effect of the continuous process). We update unit movement between local environments during each mass-extinction timestep. At these time points, each unit has a 1% chance of surviving the extinction event, but gets relocated to an adjacent local environment. These new arrivals immediately interact with their new local environment to modify the relevant niche parameter. As described above, any modifications made to a local environment’s properties persists within that local environment.

Additionally, for the purposes of demonstrating dynamics in an extremely challenging case, particle type “A” was assigned a positive production rate only within a single local environment. Hence, for type “A” to populate new local environments, A’s must “diffuse” to apply their type’s unique modification to the local niche-property. As its production rate value is 0 in any new environment, all future production of A’s in a new environment is determined by the effect of its niche construction (implemented as an increase in the value of the niche property for that type). Effectively, without “diffusion” enabled, “A” is restricted to a single local environment within the grid. In the manuscript, we provide results for such a scenario where B’s are incapable of niche construction. Here, in Figure A1, we also wish to show that an equilibrium can be reached *across the grid* when B’s also niche construct.

**Figure A1 – Diffusion, differential niche construction, and differential persistence**

This simulation is configured like Figure 3 (main manuscript) with one key difference: B now engages in niche construction with a value of +1. In Figure 3, B did not niche construct; here it does so, albeit at a weaker level than A (which has a value of +2).

**Running the Model**

The model is specified according to user-defined settings in a configuration file. Running a simulation according to the specified model requires executing the *run_*sim script with the filepath of the configuration file as its first and only command line argument (i.e., sys.argv[1]). Parameters in the configuration file are described in Table A1. The *run_sim* script uses the configuration file to construct a model object. The model is then sent to the *sim* module, which creates the environmental grid and begins computing the simulation timesteps. In doing so, the *sim* module (1) invokes a *SimEngine* module responsible for probability calculations, (2) coordinates the environment and particle objects to perform the steps required for the simulation, (3) logs the results of each timestep, and (4) formats and writes the results to file when the simulation has finished (Figure A2).

**Figure A2** – Relationship diagram depicting flow of information through interactions between the modules and classes of the ENSwR simulation to produce output.

**Table A1** – Description of all parameters comprising a configuration file.

| **Name** | **Data Type** | **Description** |
| --- | --- | --- |
| *[Dials]* |  | Header for model settings |
| seed | int | Optional declaration for random number seed |
| timesteps | int | Number of timesteps to run the simulation |
| extinction_gap | int | Timesteps between mass-extinctions |
| environment_x | int | Size of Environment grid (*x* dimension) |
| environment_y | int | Size of Environment grid (y dimension) |
| output_file | string | Filepath to direct output file |
| decay | bool | Toggle whether model includes unit decay |
| diffusion | bool | Toggle whether to include diffusion |
| infinite_resources | bool | Toggle whether to include resource limitation |
| *[Type_A/B]* |  | Header for unit types (A or B) settings |
| Types | string | Types of units (A or B) |
| A/B_niche_construction | int | Niche construction value for types |
| production_rate | int | Baseline production for types |
| residence_time | int | Timesteps to persist in decay models |
